# Biosynthesis of UV-Absorbing Mycosporine-like Amino Acids and Transcriptomic Profiling of Differential Gene Expression in Green Microalga Under Abiotic Stresses

**DOI:** 10.3390/ijms27010537

**Published:** 2026-01-05

**Authors:** Georgia Tsintzou, Evmorfia Bataka, Georgia Tagkalaki, Sofoklis Keisaris, Nikolaos Tsiropoulos, Nikolaos Labrou, Panagiotis Madesis

**Affiliations:** 1 Laboratory of Molecular Biology of Plants, Department of Agriculture Crop Production and Rural Environment, School of Agricultural Sciences, University of Thessaly, 38446 Volos, Greece; gtsintzou@uth.gr; 2 Laboratory of Biometry, Department of Agriculture Crop Production and Rural Environment, School of Agricultural Sciences, University of Thessaly, 38446 Volos, Greece; bataka@uth.gr; 3 Fresh Formula Private Limited Cosmetics Manufacturing Company, 1st km Lavriou Ave Koropiou—Markopoulou, 19400 Koropi, Greece; g.tagkalaki@freshline.gr; 4 Institute of Applied Biosciences, Centre for Research and Technology, 57001 Thessaloniki, Greece; skeisaris@certh.gr; 5 Laboratory of Analytical Chemistry and Agricultural Pharmacology, Department of Agriculture Crop Production and Rural Environment, School of Agricultural Sciences, University of Thessaly, 38446 Volos, Greece; ntsirop@uth.gr; 6 Laboratory of Enzyme Technology, Department of Biotechnology, School of Food, Biotechnology and Development, Agricultural University of Athens, 11855 Athens, Greece; lambrou@aua.gr

**Keywords:** microalgae, MAAs, abiotic stress, transcriptomic analysis, HPLC, DEGs, *Jaagichlorella luteoviridis*, UV, heat, salinity SPF

## Abstract

Microalgae display remarkable resilience to harsh environments, partly through the biosynthesis of diverse secondary metabolites. Cyanobacteria and red algae are well known to produce mycosporine-like amino acids (MAAs)—low-molecular-weight, water-soluble UV-absorbing compounds with anti-inflammatory, anticancer, and antimicrobial activities. By contrast, green microalgae typically lack detectable MAAs under standard conditions, and their responses under abiotic stress remain poorly characterized. Here, we investigated the freshwater green microalga *Jaagichlorella luteoviridis* grown under three stressors (salinity, heat, and UV) and assessed MAA induction. High-performance liquid chromatography (HPLC) revealed that stressed cultures accumulated multiple MAAs, whereas untreated controls showed no such accumulation. All stress treatments (UV, salinity, and heat) produced a substantial increase in peak intensity at 323–350 nm, whereas the control samples showed significantly lower absorption in this region. We also optimized an MAA extraction protocol suitable for “green” downstream applications in the pharmaceutical, nutraceutical, and cosmeceutical sectors and formulated an emulsion showing preliminary positive results and exhibiting an increased SPF index from 3.60 (control) to 3.78 when 0.2% MAA extract was added. Transcriptomic profiling against a reference genome revealed stress-specific differential gene expression and overexpression of specific genes of the MAA pathway, like ArioC and AroM/Aro1 SAM methyltransferases, thus identifying candidate targets for engineering enhanced MAA production. Given market demand for environmentally friendly and safe bioactives, microalgae represent a promising source of these valuable molecules.

## 1. Introduction

Microalgae is a recent term referring to the various types of aquatic organisms that have microscopic dimensions (1 μm–2 mm) and are mostly unicellular, in contrast to macroalgae [1]. Many biochemicals produced and accumulated by microalgae can be used in human food and animal feed, including nutritional substitutes, health supplements, cosmetics, medicines, biostimulants for plants, and biofuels. Especially, microalgae have been studied as a more sustainable source of antioxidants for animal feed and nutrition and livestock upgrading [2]. Microalgae applications in different industries depend on the characteristics and composition of each microalgal biomass. Recently, they have been used not only as a final product but also as ingredients with added value for downstream product development [3]. Chlorophyta are among the most promising groups for such commercial biotechnological applications, while *Jaagichlorella luteoviridis*, belonging to this group, has yet to be used for such applications.

Microalgae were the first unicellular form of plants that appeared on the planet and survived by adapting to adverse conditions and extreme environments due to the biosynthesis of various secondary metabolites, which were lost through evolution or as a result of gene mutations or silencing. Various secondary metabolites play a key role in supporting microalgal survival under abiotic stress conditions such as severe light conditions, salinity, heat, UV, etc. [4]. In addition, the significance of amino acids in the development of plants and their response to biotic and abiotic stress has garnered considerable attention in recent years. As a result of their potential practical applications, the study of amino acid metabolism and their function as sensing, signaling, and protective molecules has generated interest in both basic and applied research [5]. Although UV radiation is harmful to organisms, causing damage to biological macromolecules and even DNA, cyanobacteria and microalgae have developed strategies to be protected from UV exposure, such as through the biosynthesis of sunscreens for UV photoprotection [6].

MAAs belong to the chemical class of organic amino acids, with molecular structures resembling mycosporine. They are hydrophilic, tiny (400 Da) molecules carrying an aminocyclohexenone or aminocycloheximine ring substituted with nitrogen or imino alcohol. Their absorbance reached a maximum between 310 nm and 365 nm. In particular, 4-deoxygadusol, which acts as a precursor for MAA biosynthesis, reaches a peak with λ_max_ = 268 nm, while, until now, most known MAAs absorb at the UV region as follows: mycosporine-glycine (λ_max_ = 310 nm), palythine (λ_max_ = 320 nm), mycosporine-2-glycine (λ_max_ = 332 nm), shinorine (λ_max_ = 333 nm), and porphyra-334 (λ_max_ = 334 nm) [7]. MAAs were first observed in the 1960s as UV-absorbing pigments. In 1976, Favre-Bonvin et al. obtained a fungal metabolite associated with light-induced sporulation and published the first chemical structure of an MAA [8]. MAAs comprise a wide range of secondary metabolite groups that fulfill many purposes and exhibit additional actions in the cell beyond their well-known UV sunscreen role, which is particularly evident in some microalgal species, mainly the red ones. Substances, referred to as secondary metabolites, are generally produced by plants to make them more competitive under different environmental conditions [9].

MAAs play an important role in osmotic stress regulation in cyanobacteria and yeasts in different biological systems. Furthermore, the role of at least some of them as free oxygen radical scavengers and their key role in desiccation or salt resistance are just a couple of examples of other functions that these versatile molecules could perform. Although it is widely believed that sunscreen is an important function of MAAs, it is not yet clear whether their alternative functions are as important [10]. Various microorganisms, especially cyanobacteria, can accumulate these compounds, which are capable of absorbing ultraviolet light [11]. An abundance of these compounds has been observed in various organisms exposed to high radiation levels. The level of mycosporine synthesis can also be regulated by ambient light intensity. All of these facts suggest that in the future, MAAs could be proven to be real multipurpose substances [12]. The synthesis of these compounds is most likely carried out through the shikimate pathway, which involves amino acid synthesis of aromatic compounds, while the pentose route is also a candidate. However, the pathway has not been totally identified yet. More than 30 different chemical structures of these compounds have been identified in different prokaryotes and eukaryotes, including fungi, yeasts, microalgae, and other marine organisms such as macroalgae and corals [9].

Furthermore, in contrast to other forms of algae, such as macroalgae and Rhodophyta, the accumulation or presence of UV-induced MAAs in microalgae is not as common, and little has been reported.

Transcriptomic and bioinformatic analyses are powerful approaches for investigating differential gene expression in microalgae exposed to various environmental stress conditions [13]. Comparison of transcriptional profiles under different stress conditions (UV exposure, high temperatures, salinity conditions) and functional annotation of differentially expressed genes can lead to the identification of genes involved in the production of biotechnologically important proteins or enzymes involved in the biosynthesis of metabolites such as MAAs. Transcriptome sequencing represents an efficient and relatively cost-effective approach to acquire functional genomic information in the absence of complete genomic data [14]. Here, we report the successful development of a protocol for the increased production of MAA-like compounds from *J. luteovirides* under specific abiotic stresses, such as salinity, heat, and UV. This study outlines a successful and efficient protocol for their extraction, the identification of specific genes in the MAA pathway, and the preliminary results of an emulsion supplemented with MAA-like compounds with increased SPF values.

## 2. Results

### 2.1. Microalgal Growth Under Stresses

**Figure 1:**
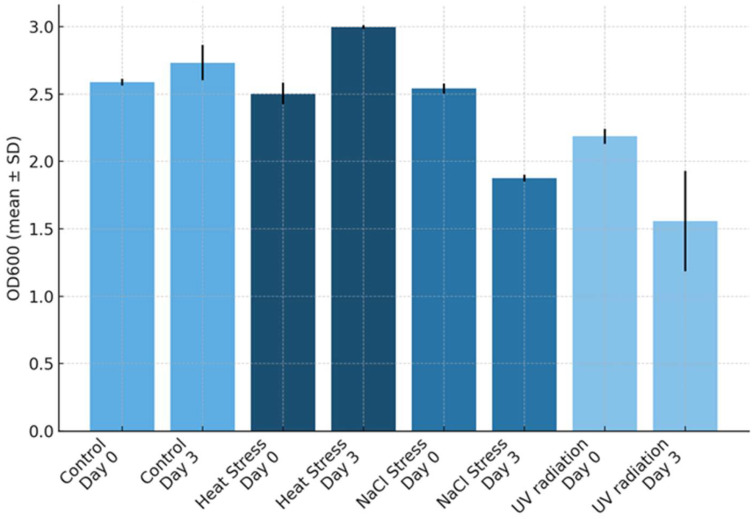
Growth results based on optical density at 600 nm (OD_600_) for control and stressed cultures (biological triplicates) at Day 0 and Day 3. Bars show mean OD_600_ ± standard deviation (SD) (presented as black lines on the columns) for each treatment and day. Treatments are distinguished by shades of blue: control, NaCl stress, UV radiation, and heat stress.

Growth responses to abiotic stress were assessed by culturing *Jaagichlorella luteoviridis* under NaCl, heat, and UV treatments alongside untreated controls for 72 h, and biomass was quantified by OD_600_. OD_600_ was measured spectrophotometrically for all cultures at baseline (Day 0) and after 72 h (Day 3). Optical density at 600 nm was used as a surrogate for cell density/biomass. Mean OD_600_ values (±SD) for control and stress-treated cultures (biological triplicates, n = 3) are presented in Figure 1, and the corresponding statistical analyses are reported in Supplementary Tables S1–S5.

Both NaCl and UV radiation stress caused a drop in OD_600_ compared to control and heat-stressed cultures, as shown in Figure 1. Control cultures exhibited the lowest variability in OD_600_ change, whereas NaCl-stressed cultures showed the greatest variability. As summarized in Figure 1, controls displayed a modest increase in cell density, while NaCl stress caused the most pronounced reduction in biomass, followed by UV stress. In contrast, heat stress at 33 °C led to an increase in OD_600_, suggesting that this microalga may be relatively heat-tolerant. A one-way Analysis of Variance for the effect of the treatments on the difference between the samples before and after the application of the stress in terms of OD_600_ values revealed a statistically significant difference in OD_600_ between at least two groups (F(3,8) = [96.65], *p* < 0.001). Tukey’s HSD Test for multiple comparisons (Supplementary Tables S2–S5) revealed that the mean value of OD_600_ was statistically significantly different between heat stress and control groups (*p*-value: 0.008), NaCl stress and control groups (*p*-value < 0.001), UV-radiation and control groups (*p*-value < 0.001), NaCl stress and heat stress groups (*p*-value < 0.001), and UV radiation and heat stress groups (*p*-value < 0). There was no statistically significant difference in OD_600_ between UV radiation and NaCl stress (*p*-value > 0.05).

### 2.2. MAAs Extraction Protocol Optimization 

To enable the development of cosmeceutical products based on MAAs, extraction procedures were optimized in accordance with green chemistry principles. Three protocols were evaluated: (i) a conventional methanol-only extraction; (ii) a mixed solvent comprising 0.5% (*v*/*v*) methanol and 0.2% (*v*/*v*) aqueous acetic acid; and (iii) a water-only method using distilled, sterile water, designed to maximize safety for human-use applications. Extracts were subsequently analyzed using UV–Vis spectroscopy.

**Figure 2:**
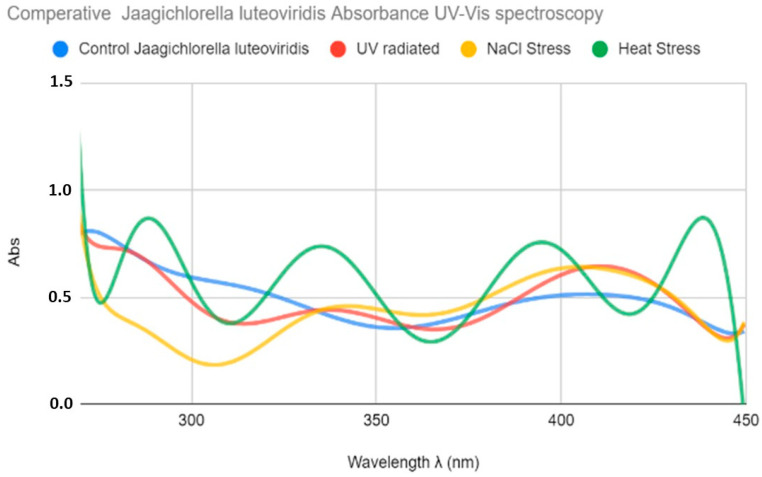
Comparative UV–Vis absorption spectra of *Jaagichlorella luteoviridis* extracts obtained from control (blue curve), UV-stressed (red), NaCl-stressed (yellow), and heat-stressed (green) cultures. All extractions were performed using methanol (MeOH) as the solvent.

The absorption spectrum of water extracts of the control as well as the treated samples of *J. luteoviridis* is shown in Figure 2. No important absorbance peaks were observed in the extracts of the control cultures. In contrast, all extracts from the treated cultures showed high and various absorbance values. Thus, a dilution of 1:10 was selected to proceed with the analysis and comparisons of results among the different treatments. Mild UV absorption with peaks at 275 and 318 nm was observed for the UV-stressed samples. However, in the visible region, a peak at 420 nm was observed in the UV-irradiated samples that was not present in the control sample. These peaks could correspond to MAA precursor molecules (275 nm) and MAA (318 nm). Regarding the salt-stressed cultures, a similar pattern was observed, with peaks at 334, 342, and about 420 nm that overlap, creating wide curves. Finally, heat-stressed cultures presented various peaks with higher intensity at (2339 and ανδ 400 nm), which are attributed to the MAA content and a peak at 437 nm in the visible area (Figure 2).

Among the three approaches, the water-only protocol produced higher UV absorbance at characteristic MAA-like compounds wavelengths, indicating that a methanol-based procedure, relying on a hazardous organic solvent, is not required. On this basis, a purely aqueous extraction was selected and is proposed here, as it reduces solvent use and volumes and streamlines downstream processing (including lyophilization) without a detectable loss in MAA yield.

### 2.3. HPLC Analysis for MAAs 

**Figure 3:**
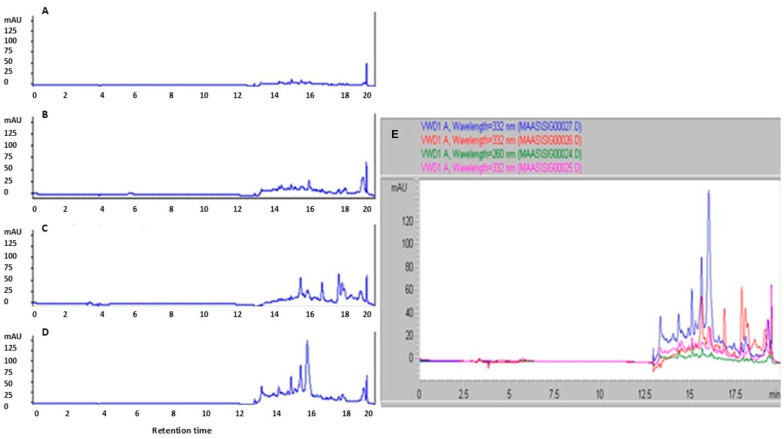
High-performance liquid chromatography (HPLC) chromatograms of extracted MAAs from *Jaagichlorella luteoviridis* cultures under different conditions: (**A**) control (with acyclovir), (**B**) UV stress, (**C**) salinity stress, and (**D**) heat stress, monitored at 332 nm. (**E**) Overlay of HPLC chromatograms showing extracted MAAs from *Jaagichlorella luteoviridis* cultures subjected to different stress conditions, monitored at 332 nm. The overlaid signals illustrate differences in MAA accumulation profiles among treatments. Retention time (RT, min) is shown on the *x*-axis, and absorbance (mAU) is shown on the *y*-axis. Also shown are control MAAs (green curve), UV-stressed MAAs (purple curve), NaCl-stressed MAAs (red curve), and heat-stressed MAAs (blue curve). The overlay demonstrates that stress-treated samples, particularly those exposed to heat, exhibit enhanced peak intensities and additional features within retention windows of 12.5–18 min, consistent with increased MAA production relative to the control.

HPLC analysis of MAA-like compound-enriched extracts from stress-treated cultures revealed prominent peaks at 332 nm (Figure 3). Acyclovir was included as an internal standard and consistently produced an intense terminal peak at a retention time (RT) of ~20 min across all chromatograms. Putative MAA-like compounds identities were assigned by comparison with published absorption maxima and retention characteristics in similar chromatographic systems [15,16,17,18,19,20]. Because commercial MAA standards are unavailable, chromatograms from UV-, salinity-, and heat-stressed samples were compared directly with those from untreated controls.

Overall, stress-treated samples exhibited higher signal intensities than controls. Peaks attributable to MAA-like compounds were consistently observed within two RT windows (2–6 min and 12.5–18 min) under the selected extraction and HPLC conditions (Figure 3) [21]. Relative to controls, all stress conditions produced multiple additional peaks in the UV range, including several features not present in the control chromatogram. Notably, many of these stress-induced peaks appeared at similar retention times across UV, salinity, and heat treatments, suggesting that *J. luteoviridis* likely synthesizes a common set of MAA-like compounds in response to different abiotic stressors.

Among the treatments, heat stress yielded the highest overall signal intensity, indicating the greatest relative accumulation of MAA-like compounds. In the absence of certified standards, a comparative assessment based on peak–height ratios supports the conclusion that heat stress leads to higher MAA-like compound concentrations than UV or salinity stress. Finally, the stability of the biosynthesized MAA-like compounds was evaluated after one month of storage at −20 °C as part of an assessment of extract robustness for downstream applications. The analysis demonstrated that the methanolic extracts remained stable over time, indicating that MAAs can be effectively preserved in methanol for at least one month without detectable degradation.

The present findings indicate that MAA biosynthesis can be enhanced in microalgae under defined abiotic stress regimes. Specifically, exposure to UV, salinity, and heat was associated with distinct chromatographic signatures and increased MAA-like signals, consistent with stress-responsive activation of the underlying biosynthetic pathway(s). This interpretation is supported by the observed overlap in retention time patterns across different stressors, suggesting that a shared set of metabolites is produced. Although the precise enzymatic steps could not be validated due to the lack of commercial standards, transcriptomic data and prior literature on absorption maxima and retention behavior provide convergent evidence that canonical MAA scaffolds (and the precursor 4-deoxygadusol) are involved.

Because no commercial MAA standards are available, identification of mycosporine-like amino acids must rely on the accepted diagnostic combination of UV–Vis spectral signatures, chromatographic behavior, and stress-induced accumulation. Across all C18 chromatograms, we observed distinct UV-absorbing peaks within the 323–350 nm region, which corresponds to the established λ_max_ range of MAAs. These peaks exhibited sharp and symmetric absorption bands, without secondary shoulders or long-wavelength absorbance, thereby excluding interference from phenolic or flavonoid compounds, which characteristically show broader spectra with multiple maxima extending beyond 380–400 nm. In contrast, a non-MAA peak at about 415 nm, a profile typical of complex pigmented metabolites, did not overlap with the putative MAA retention-time window, confirming that our spectral resolution can discriminate MAA-type from non-MAA chromophores [21,22,23,24].

Chromatographically, the UV-active peaks eluted consistently within RT 12.5–18 min, a retention time region widely reported for highly polar MAAs under comparable C18 conditions [21,22,23]. Although HILIC analysis did not reveal strong MAA-like signals, the C18 method successfully detected multiple hydrophilic, UV-absorbing metabolites whose retention times match published values for porphyra-334, shinorine, palythine, and related compounds. The LC–MS data did not yield database matches for MAAs, an expected outcome given that MAAs are not represented in commercial MS libraries and ionize poorly under generic positive-ion conditions, yet importantly, no masses corresponding to phenolic or flavonoid structures were detected in the 323–350 nm RT window.

A biologically coherent pattern further supports MAA identity. All stress treatments (UV, salinity, and heat) produced an increase in peak intensity at 323–350 nm, whereas the control samples showed significantly lower absorption in this region. This stress-inducibility is a hallmark of MAA-like compound biosynthesis, reflecting the activation of photoprotective pathways derived from the shikimate and pentose-phosphate routes. To strengthen spectral specificity, we additionally calculated UV–Vis absorbance ratios (A_350_/A_310_ and A_330_/A_290_), following the precedent of ratio-based MAA validation approaches used in the literature (see Supplementary Table S6).

In analogy to the absorbance ratio method commonly applied in MAA research [25], we calculated spectral indices, defined as ABS(λ_max_)/ABS(_450nm_). This ratio enhances the contrast between the dominant MAA-like absorption band and the broader background contributed by phenolic or flavonoid chromophores (approx. 450 nm). As such, it provides an additional, ratio-based criterion for discriminating MAA-type spectra from other metabolites. The calculated indices (Supplementary Table S2) demonstrate that the increase in ABS(λ_max_)/ABS_450_ under UV, salinity, and heat stress is consistent with the induction of mycosporine biosynthesis relative to the control condition.

Taken together, including (i) diagnostic UV maxima, (ii) narrow-band spectral shape, (iii) appropriate retention times, (iv) absence of phenolic/flavonoid-like spectral features, (v) stress-dependent accumulation, and (vi) supportive UV–Vis ratio analysis, our results provide multi-criterion evidence for the presence of MAA-type metabolites in stressed microalgal extracts, despite the current unavailability of authentic standards.

### 2.4. Formulation, Development, and Test of Cream Supplemented with MAA

Emulsion creams supplemented with 0.1% and 0.2% (*w*/*w*) MAA extracts from *Jaagichlorella luteoviridis* were formulated. Stability was assessed over 3 months under accelerated conditions. Based on the accelerated stability tests (Supplementary Tables S7 and S8), the initial physicochemical characteristics of the base cream were not affected by the addition of MAA extracts at either concentration (0.1% or 0.2% *w*/*w*). Both MAA formulations were stable for 1 month at all temperatures tested. After 2–3 months at 50 °C, only minor signs of instability were observed, with the 0.1% MAA cream exhibiting slightly greater stability than the 0.2% formulation. At 40 °C for 3 months—which, under our accelerated protocol, corresponds to approximately 2 months of real-time shelf life—both creams remained stable. These results suggest that incorporation of an MAA-like compound does not compromise product integrity and support the feasibility of a marketable formulation once safety and allergenicity tests are positive. Both MAA-like compound-supplemented emulsions remained stable and were comparable to the unsupplemented control with respect to pH, viscosity, homogeneity, and phase separation upon centrifugation.

The MAA-like compound-supplemented emulsions were also evaluated regarding their sun protection factor (SPF) to assess their potential application as natural UV-protective formulations. SPF determination was conducted spectrophotometrically according to standard in vitro methodologies. Both MAA-like compound-enriched emulsions exhibited a measurable increase in SPF compared to the control formulation lacking MAA extracts, demonstrating the possible photoprotective efficacy of the incorporated compounds (Table 1). Notably, the emulsion containing 0.2% (*w*/*w*) MAA-like compounds extract showed a more pronounced enhancement in SPF value, suggesting a concentration-dependent effect at *p* < 0.001. These findings confirm that MAAs from *Jaagichlorella luteoviridis* can act as effective, biocompatible UV filters, reinforcing their potential suitability for inclusion in cosmeceutical sunscreen formulations, provided the in vitro and in vivo UV protection, safety, and allergenicity tests are positive. However, we have to stress that the Mansure test is a crude test, and thus the results presented here are preliminary concerning the development of a marketable product.

### 2.5. Transcriptomic Analysis

To identify transcriptional responses across experimental conditions, differential expression analysis was performed using the DESeq2 package in R. Input data consisted of transcript-level quantifications generated by Salmon, which were then imported and processed within DESeq2 for normalization and statistical testing. As Salmon produces transcript-level abundance estimates, each transcript was mapped to its corresponding putative gene or ortholog using EggNOG-mapper annotations. This allowed for biological interpretation at the gene level, even though a de novo transcriptome reference was used.

**Figure 4:**
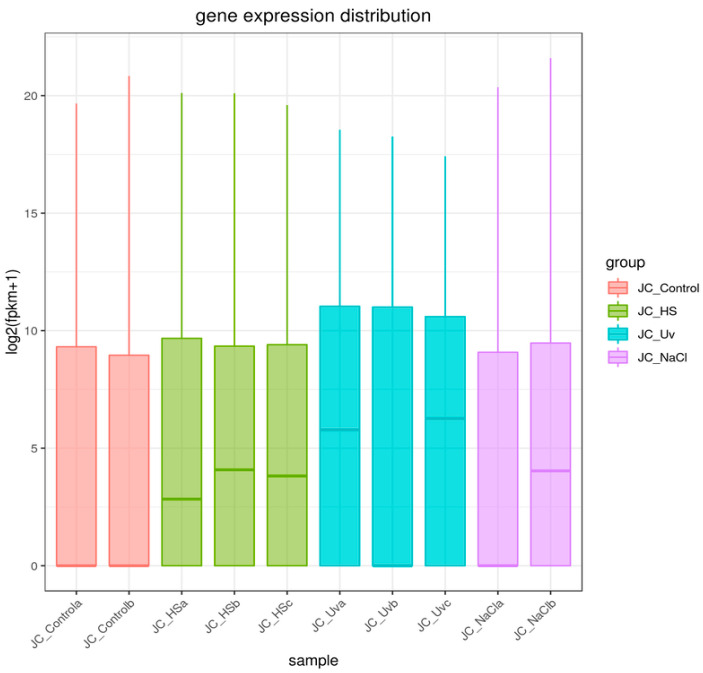
Sample gene expression distribution box plot. The *x*-axis represents the name of the sample, and *y*-axis indicates log_2_(FPKM + 1). Parameters of box plots are indicated, including maximum, upper quartile, mid-value, lower quartile, and minimum. Thick horizontal line inside each box present the median (50th percentile) of the data. Thin vertical lines extending above and below the box present the maximum values within 1.5 × IQR from the quartiles.

**Figure 5:**
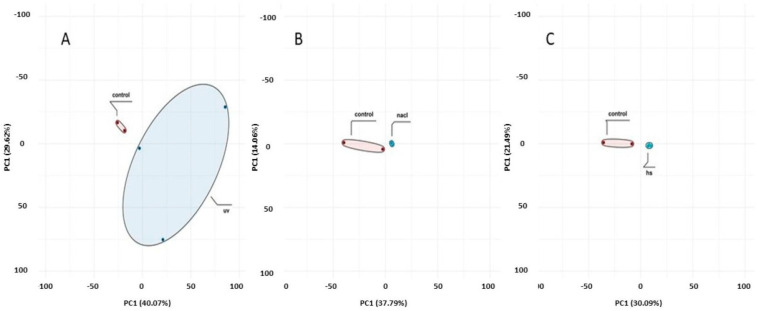
Principal component analysis (PCA) of transcriptomic profiles. PC1 and PC2 explained (**A**) 69.7% (UV vs. control), (**B**) 51.9% (NaCl vs. control), and (**C**) 51.6% (HS vs. control) of the variance.

To compare transcriptional profiles across conditions, gene expression was quantified as FPKM and summarized per sample using boxplots (Figure 4). For biological replicates, the final FPKM for each gene was computed as the arithmetic mean across replicates. Global sample relationships were visualized via principal component analysis (PCA) of the expression matrix (Figure 5). Principal component analysis (PCA) revealed treatment-dependent shifts in global transcriptional profiles (Figure 5). PC1 and PC2 explained 51.6% (HS vs. control), 51.9% (NaCl vs. control), and 69.7% (UV vs. control) of the variance. While the separation between treated and control samples was most pronounced under UV exposure, a substantial proportion of the total variance resides in higher PCs. Therefore, PCA patterns should be interpreted as indicative of broad expression shifts rather than complete transcriptomic partitioning.

**Figure 6:**
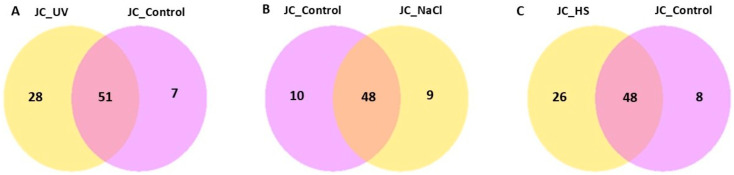
Co-expression Venn diagram illustrates the number of genes that are uniquely expressed within each group/sample, with the overlapping regions showing the number of genes that are co-expressed in two or more groups/samples for (**A**) UV vs. control, (**B**) NaCl vs. control, and (**C**) HS vs. control.

**Figure 7:**
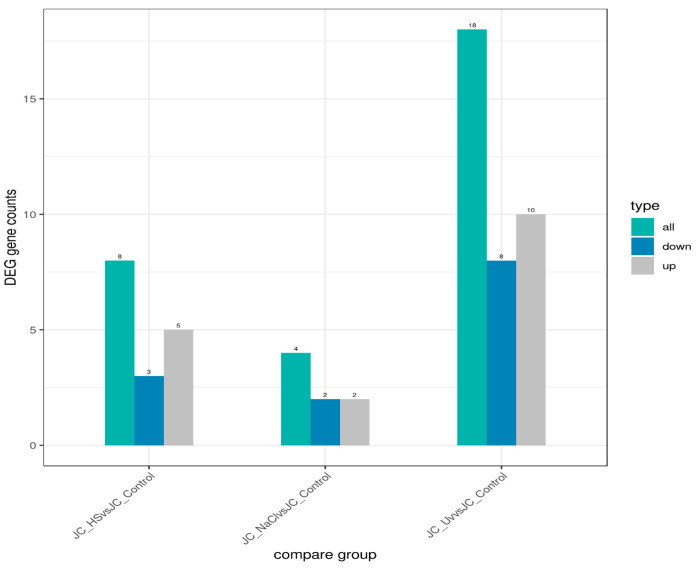
Differential gene expression statistics. Histogram shows the number of differentially expressed genes. Blue bars represent up-regulated genes, and gray bars represent down-regulated genes. Numbers above each bar indicate the total count of genes in each category.

Condition specificity and overlap of expressed genes were evaluated using a co-expression Venn diagram (Figure 6), which reports the number of genes uniquely expressed in each group as well as those shared among two or more groups. Differential expression outcomes are summarized in Figure 7, which presents, for each pairwise comparison, the total number of differentially expressed genes (DEGs) together with the counts of up-regulated and down-regulated genes.

**Figure 8:**
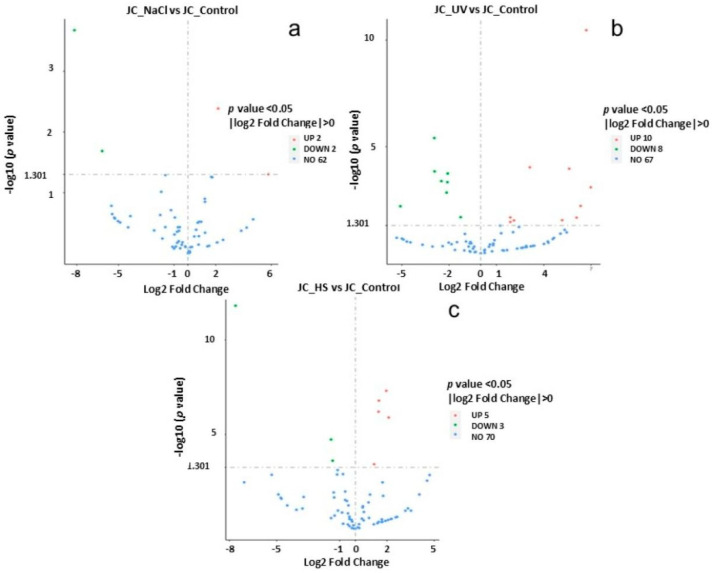
Differential gene volcano map. (**a**) NaCl vs. control, (**b**) UV vs. control, and (**c**) heat vs. control. The abscissa in is log_2_FoldChange, and the ordinate is -log10*p*adj or -log10*p*value. The blue dashed line indicates the threshold line for differential gene screening criteria (**a**–**c**).

Volcano plots (Figure 8) were used to infer the overall distribution of differentially expressed genes. In Figure 8, the *x*-axis shows the fold change in gene expression between different samples, and the *y*-axis shows the statistical significance of the differences. Red dots represent up-regulation genes, and green dots represent down-regulation genes.

**Figure 9:**
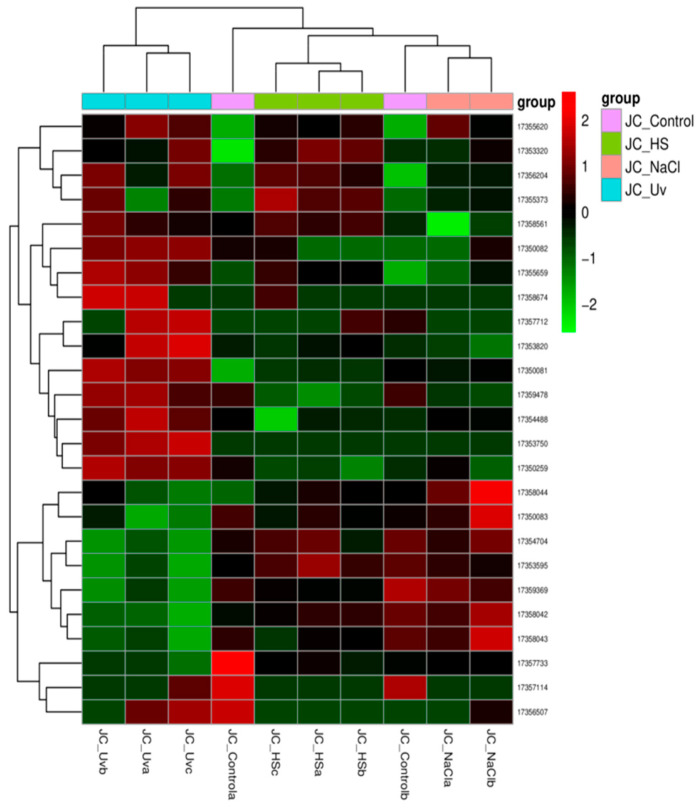
Differential expression gene clustering heatmap.

Figure 9 shows the heat map of all the differentially expressed genes in the comparison group, which were pooled as the differential gene set. For more than two groups of experiments, cluster analysis can be carried out on different gene sets, and genes with similar expression patterns can be clustered together. We used the mainstream hierarchical clustering to cluster the FPKM values of genes and homogenized the row (Z-score). The genes or samples with similar expression patterns in the heat map were gathered together. The color in each grid reflects not the gene expression value, but the value obtained after homogenizing the expression data rows (generally between −2 and 2). Therefore, the colors in the heat map can only be compared horizontally (the expression of the same gene in different samples), but not vertically (the same sample). There is not only inter-group clustering, but also inter-sample clustering.

The overall results of FPKM cluster analysis, clustered using the log_2_(FPKM + 1) value, are shown in Figure 9. Red color indicates genes with high expression levels, and blue color indicates genes with low expression levels. The color ranging from red to blue indicates that log_2_(FPKM + 1) values range from large to small.

To gain insight into the biological processes affected by the different stress conditions, particularly the production of MAAs, pathway enrichment analysis was performed based on differentially expressed transcripts. The analysis was conducted using the clusterProfiler package in R, leveraging KEGG ortholog (KO) identifiers obtained from EggNOG-mapper annotations. Prior to enrichment, only transcripts that passed differential expression thresholds (adjusted *p*-value < 0.05) were retained. KEGG pathway enrichment was first applied globally across all available pathways, allowing for an unbiased assessment of biological responses. From this broad analysis, attention was then restricted to pathways or gene sets relevant to MAA biosynthesis. Candidate pathways were identified using keyword-based filtering (e.g., “shikimate”, “aminobenzoate”, “UV”, “aromatic amino acid metabolism”), as well as manual review of enriched pathway descriptions. In parallel, a complementary approach was taken in which EggNOG annotations were directly scanned for MAA-related terms at the transcript level. This shortcut allowed rapid identification of transcripts potentially involved in MAA biosynthesis or photoprotective responses without relying solely on statistical enrichment.

**Figure 10:**
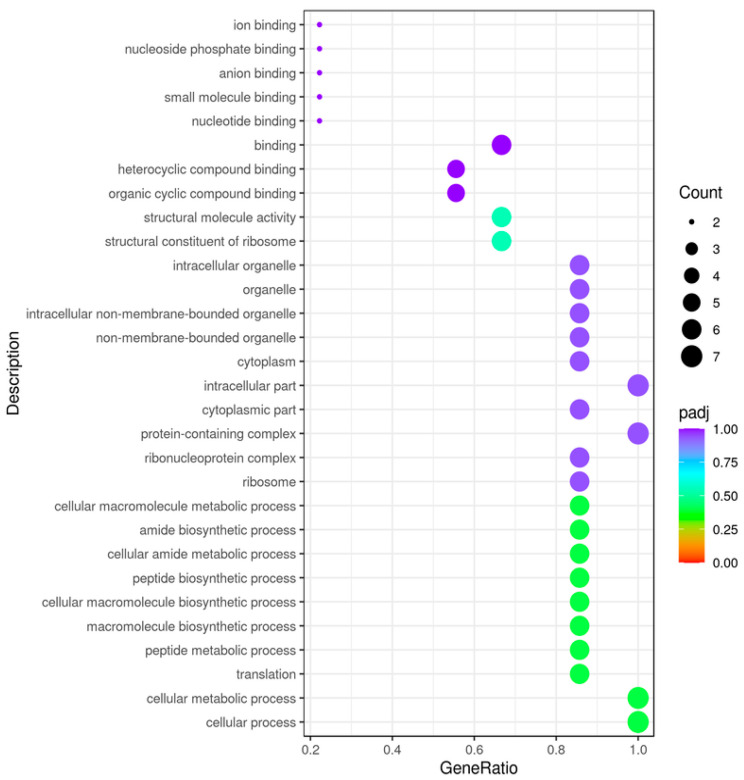
GO enrichment analysis scatter plot. JC_Uv vs. JC_Control. The abscissa in the graph is the ratio of the differential gene number to the total number of differential genes on the GO Term, and the ordinate is GO Term.

The GO enrichment analysis result suggests that the most significant variation refers to UV stress and is drawn according to major categories of biological processes (BP), cell components (CC), molecular functions (MF), and categories of up- and down-regulated genes (Table 2). Higher values correspond to higher significance. The different colors represent the three GO subclasses of BP, CC, and MF. In Figure 10, the abscissa is the ratio of the number of differential genes linked to the GO Term to the total number of differential genes, and the ordinate is the GO Term. The size of a point represents the number of genes annotated to a specific GO Term, and the color from red to purple represents the significance level of the enrichment.

Furthermore, in the UV vs. control comparison, we identified several differentially expressed genes directly related to the shikimate pathway and downstream aromatic metabolism. A transcript annotated as chorismate synthase (aroC)—NODE_14408_g493_i22 (log_2_FC og_2_FCDE_*p*adj ≈ 0.004)—and multiple transcripts corresponding to the functional AROM/ARO1-like enzyme, such as NODE_1259_g0_i766 (log_2_FC og_2_FCult*p*adj < 1 × 10^−5^), were among the most strongly induced genes. In addition, UV stress triggered a broad induction of SAM-dependent O-methyltransferases (EC 2.1.1.*), including highly up-regulated transcripts such as NODE_17774_g4869_i0 and NODE_18650_g38_i46, both annotated with multiple methyltransferase PFAM domains. By contrast, a glutathione S-transferase zeta 1 (GSTZ1)-like transcript—NODE_14392_g0_i5482 (log_2_FC ≈ −26.1, *p*adj ≈ 1 × 10^−6^)—was strongly down-regulated under UV, NaCl, and heat stress, indicating a possible rerouting of aromatic amino acid flux away from degradation and toward secondary metabolite and/or MAA biosynthesis. In addition, under NaCl and heat stress, we detected differential expression of several SAM-dependent methyltransferases, including protein and small-molecule O-methyltransferases such as NODE_12635_g1_i3 (NaCl) and NODE_20949_g1_i0 (heat), as well as mild induction of aroC or ARO1 orthologs. Nevertheless, the consistent and very strong down-regulation of the GSTZ1-like transcript NODE_14392_g0_i5482 across all three stresses (log_2_FC ≈ −23 to −26) suggests that aromatic amino acid catabolism is broadly repressed, which may favor the accumulation of upstream precursors that can be diverted into photoprotective pathways such as MAAs (see Supplementary Figure S1).

## 3. Discussion

*J. luteoviridis* proved to be heat-resilient at 33 °C, and thus could be proposed as the selected organism for sustainable agriculture and aquaculture in the era of climate change, while it has to be tested to determine its thermo-extremophile properties in future research.

Cultures grown under normal conditions did not contain any UV-absorbing compounds when UV–Vis spectroscopy was performed. However, after exposure to the three different abiotic stress regimes, microalgal cells produced various compounds that absorbed in the UV region. Previous research has shown that some microalgae in the class of Chlorophyceae produce UV-absorbing compounds; however, they have different absorption maxima and low concentrations. The presence of these compounds is assumed to be more widespread in the Chlorophyceae class, but there may be limitations to how many of these compounds can be produced, depending on the physiological characteristics of the organism and its environment [17]. It seems that all the tested abiotic stresses can cause MAAs production as a quick response. HPLC-DAD analysis demonstrated that MAA-like compounds are indeed present in the crude MAA-like compound mixture when abiotic stress is applied to the culture. UV radiation may cause harmful effects on various photosynthetic organisms. Some plants, though, such as green algae, have developed mechanisms to counteract these effects. These mechanisms include the production of UV-absorbing compounds and methods to repair any such damage. It was previously considered that higher organisms did not possess this pathway and that they would accumulate these molecules in small quantities and only from their diet. However, recent studies have shown that some higher organisms possess genes involved in the shikimate pathway. In addition, many mycosporine compounds found in nature can be produced by various late biosynthetic steps. These include the addition of free amino moieties to the basic molecular core [26,27]. Llewellyn et al., in 2010, suggested that a deeper understanding of these functions should also take into account lesser-known aspects, while new evidence is accumulating that MAAs may have additional functions, for instance, serving as antioxidant molecules, scavenging toxic oxygen radicals, and compatible solutes following salt stress [27]. Their production is found to be induced by desiccation, heat, or cold stress in some specific organisms. MAAs have also been suggested to function as accessory light-harvesting pigments during the process of photosynthesis as an intracellular nitrogen reservoir, and they were first involved in fungal spore-based reproduction [27]. The catalyzed transition of double bonds within a molecule results in broad absorption bands ranging generally from 310 to 360 nm. For MAAs with structures that are based on the cyclohexenone ring, the bands present a range from 280 to 320 nm for the maximum peak wavelength in the UV-B region. In parallel, for structures based on a cyclohexenimine core, the bands range from 320 to 400 nm in the UVA region. Changes in the maximum absorption are often caused by the substituent effects of the molecules when it comes to big MAA diversity [19]. Adjusting the growth conditions of the microalgal cultures (temperature, salinity, nitrogen concentration, and exposure to UV) after the exponential phase resulted in overexpression of MAAs. However, this method involves subjecting microalgae to stressful conditions, which can negatively impact their cell division and thus ultimately reduce the overall yield of biomolecules [19].

The production of MAAs by various microalgae can also serve as a defense mechanism against ultraviolet radiation. It allows organisms to capture photons without interacting with their cellular machinery, such as DNA and proteins. For MAAs, the catalyzed transition of double bonds within their molecules results in broad absorption bands ranging from 310 to 360 nm. For MAAs with structures based on the cyclohexenone ring, the bands present a range of 280 to 320 nm for the maximum peak wavelength in the UVB region. In parallel, for structures based on the cyclohexenimine core, the bands range from 320 to 400 nm in the UVA region. Changes in the absorption maxima are often caused by substituent effects of the molecules with respect to a large MAA diversity [28,29].

The use of marine-derived antioxidants, specifically MAAs, as potential anticancer agents is supported by their anti-proliferative effects on neoplastic cells. Furthermore, MAA has been reported to promote wound healing in HaCaT cells [30]. For instance, another strain, *Chlamydomonas hedleyi*, was reported to possess UV-absorbing MAAs, which protected human keratinocytes from the harmful effects of UV radiation by influencing the expression of genes linked to oxidative stress, inflammation, and skin aging caused by UV exposure [31].

An HPLC analysis method was developed to provide a more accurate assessment of their diversity and concentration in aquatic organisms. This method is selective enough to identify most MAA-like compounds, including highly polar compounds such as shinorine, mycosporine-2-glycine, and palythine-serine, as well as medium- and low-polarity compounds [32]. A new method for the efficient determination of eleven algal MAA-like compounds was developed using ultrahigh-performance liquid chromatography (UHPLC) with diode-array detection (DAD), enabling the identification and quantification of these compounds in a variety of red algae samples with shorter analysis times than traditional techniques [32].

Macroalgae and Rhodophyta are known to produce MAAs; however, the accumulation or presence of UV-induced MAAs in green microalgae is not as common, and little has been reported. Therefore, the application of various stress factors such as heat, UV, and salinity to microalgal cultures suggested that the synthesis of MAAs increases under specific abiotic stresses. This could be attributed to the expression of genes involved in the MAA biosynthesis pathway. Furthermore, optimizing these stress factors and lab-scale conditions resulted in an increase in the yield of MAAs, including in the green microalgae being studied in the present study. Interestingly, the results presented here were achieved in a more sustainable manner in terms of cellular biomass content. Thus, UV, salinity stress, and heat stress maintained and even increased biomass yield while achieving greater MAA accumulation.

The sustainable approach adopted in this study suggests that mild heat stress application did not cause any loss of biomass, in contrast to salinity and UV stress. However, biomass yield increased significantly during heat stress, along with enhanced MAA-like compounds biosynthesis. These results showed that green, freshwater *J. luteoviridis* microalgae have extremophilic properties and heat resilience, suggesting that they could be a great choice for cultivation in the era of climate change in countries with high temperatures. In parallel, the selection of a water-based extraction protocol for MAA isolation proved to be successful, providing an opportunity for biorefinery extraction. This study also revealed that the aquatic extracts of *J. luteoviridis* could be promising compounds for cosmeceutical applications, being, at the same time, green and safe bioactive ingredients. These extracts, specifically those resulting from heat stress, are rich in MAAs, which are known for their anti-aging, anti-inflammatory, and antioxidant properties. Further research on the purification and isolation of these compounds is anticipated to lead to their characterization and, possibly, to the exploitation of novel compounds.

The MAA-like compound extracts, when tested in emulsion creams, showed their potential to act as anti-UV sun-protecting creams, as they exhibited increased SPF index, suggesting enhanced UV absorbance, especially when they were added at a 0.2% *w*/*w* in the cream, which is the standard for the industry. It is also important to notice that the products were stable in an accelerated stability test, suggesting that the products could easily go to the market. However, the Mansure methodology used is a crude test, suggesting that the results should be considered as preliminary, although promising [11].

According to evidence, most MAAs in autotrophic eukaryotes and other prokaryotic organisms are produced via the shikimate pathway. It was previously believed that higher organisms did not possess this pathway, and they would accumulate these molecules only from their diet in small quantities. Recent studies have shown that some higher organisms do possess genes that are involved in the shikimate pathway. The large number of mycosporine-like compounds found in nature could be produced by various late biosynthetic steps. These include the addition of free amino-moieties to the basic molecular core [27]. In *Anabaena variabilis*, o-methyltransferase was found to be part of both the pentose phosphate and shikimate pathways.

In conclusion, the results obtained in this study indicate that *J. luteoviridis*, when subjected to abiotic stress conditions, has significant potential for the production of valuable secondary metabolites, such as MAAs, which could be used in pharmaceutical and cosmeceutical products. This study demonstrated the ability of *J. luteoviridis* to produce MAAs only through the application of abiotic stresses such as UV radiation, heat, and salinity stress. Among these treatments, heat stress showed the best results for UV-absorbing content, cultural cellular gain, and high HPLC peaks for MAAs. This research proposes *J. luteoviridis* as a heat-resilient strain that can be cultured in warm environments, resulting in higher biomass yields. We also showed that it is important to carefully evaluate and select the stress application for the targeted microalgal species and desired metabolites, as the bioavailability of many compounds in the extract depends on downstream processes.

## 4. Materials and Methods

### 4.1. Microalgal Culture and Growth Conditions

*J. luteoviridis* strain 211/5Β was obtained from the Culture Collection of Algae and Protozoa (CCAP-SAMS Limited, Oban, Scotland, UK). The experimental setup included four levels of treatment, with the control as the first level, cultured in normal conditions, and stress levels, each treated with an abiotic stress factor (UV irradiation, salinity, and heat stress), with three biological replicates each. All cultures were inoculated from the same initial unialgal culture of *J. luteoviridis* and were maintained in Erlenmeyer conical flasks with a 1 L volume, carrying a silicon cap with a single hole 5 mm in diameter and a silicon, transparent air tube with a 4–6 mm outer diameter. All the materials were autoclaved at 122 °C for 20 min. Blue-Green BG-11 medium was selected as the most appropriate growth medium for *J. luteoviridis* and prepared according to directions, with a slight modification in enrichment with Trace Elements (TE) Stock 8 in 1 mL/1 L of media. BG-11 (Blue Green) media containing the following nutrients per L: 10 mL from each one of eight stock solutions, all with an initial volume of 500 mL, containing 75 g of NaNO_3_, 2 g of K_2_HPO_4_, 3.75 g of MgSO_4_·7H_2_O, 1.80 g of CaCl_2_·2H_2_O, 0.30 g of Citric acid, 0.30 g of ammonium ferric citrate green, 0.05 g of Na_2_EDTA, and 1 g of Na_2_CO_3_, respectively, autoclaved for 20 min at 122 °C and stored in the refrigerator [33]. The light intensity was constant at 26 ± 2 µmol m^−2^ s^−1^, with a light: dark cycle of 12:12 h inside a plant growth chamber at a room temperature of 24 ± 1 °C with a humidity level of 65% [34]. The air supply was constant and continuous, and the flasks were stirred twice a day to achieve culture homogeneity and avoid shelf cell shading. The achievement of unialgal cultures was verified via light microscopy using a Leitz Laborlux 12 pol with 50× magnification on a weekly basis. The optical density at 600 nm (OD_600_) was measured twice a week using an Eppendorf Biophotometer (6131, Marysville, WA, USA). When the OD_600_ reached 2.9, meaning the stationary state marginally began, BG-11 medium with 1% agar was solidified in sterilized Petri dishes and streaked with enriched culture samples. The dishes were incubated under the same conditions for two weeks and kept as stock cultures at 4 °C for one year with great revival ability. In parallel, at a 3.0 OD_600_ value, abiotic stress conditions were applied to the experimental units, and the experiment lasted three days [35]. In order to select the levels of the stresses used here, we performed pilot dose–response assays in *Jaagichlorella luteoviridis*. Lower stress intensities (0.5–1.0 M NaCl, UV < 10 W, temperature ≤ 30 °C) resulted in minimal metabolic responses and negligible UV absorbance changes. Conversely, more severe conditions caused extensive growth inhibition and loss of viability. The selected levels represent strong but sub-lethal stresses, allowing metabolic reprogramming while retaining adequate biomass, consistent with the aims of the study.

### 4.2. Abiotic Stresses Protocols 

As for the UV setup experiment (the second level of treatment), 300 mL of the three conical flasks was transferred to open Petri dishes with a diameter of 90 mm and homogenized daily by pipetting up and down to avoid the shelf shading phenomenon while irradiated with UV light for three days, and the light–dark ratio was 12:12 h [36]. The UV light source was 20 W, 220 V, with a UV λ_max_ of 263 nm. Microalgal optical density was measured daily to assess adaptation. For the third level of treatment, heat stress, three cultures of 200 mL each, with an OD_600_ reaching 3, were kept in an incubator with shaking aeration and the same light conditions as the controls. Initially, the temperature was set at 29 °C for 24 h to provide time for adaptation to the cells, and then at 33 °C for the remaining 2 days [36]. For salinity stress, a final volume of 300 mL of *J. luteoviridis* culture with an OD at 600 nm reaching 3 and a final concentration of NaCl at 2 M was set up in triplicate in the growth chamber under the same conditions as the controls. This high salinity concentration was previously reported to induce MAA production when implemented in cyanobacterial cultures [37]. Upon application of stress, OD_600_ was measured daily to evaluate the cultural gain or loss in cellular biomass. The selected stress levels (2 M NaCl, UV-C 20 W at 263 nm, and 33 °C) were based on preliminary range-finding experiments designed to identify sub-lethal exposures that consistently induce physiological stress without causing culture collapse, in agreement with reported tolerance thresholds for chlorophyte microalgae. Minor differences in OD_600_ at Day 0 reflect measurement variability among biological triplicates and do not represent treatment effects, as all cultures were inoculated from the same starter culture prior to stress application [38,39,40,41].

### 4.3. Statistical Analysis

One-way Analysis of Variance was performed to compare the effects of the treatments on the difference between before and after stress OD_600_ values and the SPF index. Moreover, Tukey Post hoc tests were performed. The analysis was carried out using R version 4.3.2. [42]. The package used to produce the graphs was ggplot2 [41,42]. All cultures were inoculated from a common exponentially growing stock culture at the same target cell density. However, minor differences in OD at Day 0 arose from biological variability among replicate flasks, as well as intrinsic measurement noise associated with dense microalgal suspensions. These variations were small (within ±0.1–0.15 OD units) and fell within the expected biological and technical limits for microalgal OD-based quantification.

### 4.4. MAA Extraction Protocol

*J. luteoviridis* cells were harvested via centrifugation at 4000 rpm for 4 min at 6 °C (in a Frontier FC5916/R, Ohaus, Parsippany, NJ, USA), and the pellets were frozen at −20 °C. Overnight lyophilization took place (in a Lyoquest -55 plus, Telstar, Barcelona, Spain), and samples were stored in tubes under dry and dark conditions before proceeding with MAA analysis. To align with the principles of downstream processing (DSP) for cost-effective protocols with high yields and to extend them to the production of UV-absorbing secondary metabolites such as MAAs, three protocols were tested to detect differences in MAA yields. The first protocol utilized methanol as the extraction solvent [5,17]. The second protocol utilized a solution with 0.5% methanol and 0.2% aqueous acetic acid (*v*/*v*) [43], while the third protocol used only H_2_O as an eco-friendly alternative for the MAAs extraction [28]. Freeze-dried samples of approximately 20 mg dry weight were transferred into 2.0 mL Eppendorf tubes and subjected to extraction. Firstly, 1000 μL of distilled sterile water was added to rehydrate the samples for 5 min, followed by vigorous vortexing. Subsequently, treated samples, in addition to the controls, were extracted via overnight incubation at 4 °C in a proper solvent as per protocol to maintain the extracellular water-soluble MAA content. Next, centrifuging at 9000 rpm for 8 min was performed, and the supernatants were transferred into new 1.5 mL Eppendorf tubes. Thereafter, these extracts were immediately analyzed using UV–Vis spectroscopy (Spectrophotometer V-630BIO, Jasco, Tokyo, Japan) to confirm the existence of absorbance peaks in the UV area, indicative of the MAAs content. Owing to the specific MAAs extraction protocol and literature evidence, all peaks in the UV region were attributed to the MAA content [44]. The supernatants were evaporated in the cases of methanol under vacuum at 45 °C using a rotary evaporator (RV3 Eco, IKA, Staufen, Germany) and re-dissolved in injection water, followed by UV–Vis spectroscopy and lyophilization.

### 4.5. MAAs HPLC Analysis

Completely dried extracts were mixed with 1000 μL of HPLC-grade methanol and vortexed for 30 s for thorough dissolution for column safety and sustainability. All samples were filtered utilizing 0.2 μm syringe filters prior to HPLC analysis and an HP 1100 liquid chromatograph (Hewlett-Packard GmbH, Waldbronn, Germany) equipped with a ternary-delivery system, a variable-wavelength UV detector, and a HP ChemStation LC 3D chromatography manager data. A C18 column was used, along with a guard column. The flow rate was set at 1 mL·min^−1^. The temperature of the column oven was set at 40 °C. The mobile phase consisted of A: acetonitrile with 0.1% (*v*/*v*) formic acid, B: water with 0.1% formic acid, and C: methanol: water 70/30, and all solvents were of HPLC grade and ultra-pure water. The total runtime was 20 min (B: 0–15 min, A: 15–18 min, C: 18–20), the detection wavelength was set at 332 nm, and the injection volume was 20 μL each time with 1:10 acyclovir as an internal standard. MAAs were identified by comparing previously published information on the absorption spectra and retention times. Moreover, the stability of the biosynthesized MAAs molecules was checked after a period of time (one month) [10]. Owing to the absence of standard MAAs solutions in the market, all four HPLC chromatograms obtained from the partially purified MAAs of the control-untreated cultures with the UV, salinity, and heat stress-treated samples were compared and showed significant differences. Moreover, acyclovir was used as an internal standard, with an intense peak at RT of 20 min at the end of all the chromatograms. Based on the specified MAA extraction protocol, all the peaks were attributed to the UV-absorbing MAA content [15].

### 4.6. Formulation and Development of Cream Supplemented with MAA

Regarding the main stability results of the three different potential algae extract carriers that were developed in the past months (a water-in-oil (W/O) emulsion cream/an oil-in-water (O/W) emulsion cream/an aqueous gel), it was decided to proceed with an oil-in-water (O/W) emulsion cream and an aqueous gel. Both bases were formulated using 0.1% and 0.5% (*w*/*w*) of glycerine-aqueous extracts derived from *J. luteoviridis*. For all measurements, a cream without extracts was used as a control.

The procedure for the preparation of oil/water emulsion was as follows: the oily phase (Cetearyl Alcohol, Sodium Cetearyl Sulfate, Caprylic/Capric Triglyceride) and the aqueous phase (water, glycerine, Polyacrylate Crosspolymer-6, sodium gluconate) were heated separately to 80 °C. Then, the oily phase was stirred into the water phase, and intensive agitation followed using a Silverson mixer. The system was maintained under constant stirring until it cooled down to 35 °C. Subsequently, phenoxyethanol (1% *w*/*w*) and the microalgae extracts (microalgae extract (MAA) 0.1% and 0.2% *w*/*w*) were added, and the emulsion was uniformly mixed.

Afterward, product stability testing was carried out in order to determine any changes that may occur in the characteristics of the subjective samples more rapidly than would be expected in “normal” conditions of storage. Using plastic containers suitable for cosmetics, approximately 60 g of samples was exposed to different storage conditions, such as high and low temperatures (50 °C, 45 °C, 40 °C, 50 °C), room temperature, and daylight (UV). A full product stability needs typically 3 months to be completed, and the samples were checked at the end of each month. Physical and chemical properties were evaluated in samples packaged in a suitable container at the initial time and after the 1st, 2nd, and 3rd months of storage using the following methods: (1) pH: The pH was measured using a pH meter, Denver instrument UB-S, equipped with a glass electrode, directly into the samples. (2) Analytical centrifugation (only for face cream samples). Samples (5 g) were centrifuged at 3000 rpm for 2 h (1 h, rest for 30 min, and repeat for 1 h), and the eventual phase separation was analyzed in order to assess their stability during aging. (3) Viscosity: The viscosity was determined using a Brookfield viscometer using S95 at 1.5 rpm. (4) Homogeneity: the samples were tested for homogeneity by their visual appearance and touch affinity assessment. Physical and chemical properties, including measurements of pH, color, and phase separation, were evaluated in formulations packaged in suitable plastic containers. These characteristics were observed at 25 °C, 40 °C, 45 °C, 50 °C, 55 °C, and daylight (UV) at D0 and the 1st, 2nd and 3rd month of storage. The accelerated stability centrifugation test is of high interest since it provides a quick estimate of stability properties.

### 4.7. Determination of Sun Protection Factor (SPF) of Sunscreens via UV Spectrophotometry

The emulsion was tested for the sun protection factor (SPF) according to Mansur et al. (1986) [45], utilizing UV spectrophotometry and the following equation:

$${\mathrm{SPF}}_{\mathrm{spectrophotometric}}=\mathrm{CFx}*\sum_{290}^{320}ΕΕ\left(\lambda \right)*I\left(\lambda \right)*Abs(\lambda )$$

CF = 10 (Correction factor)

EE(λ)x Ι(λ): constants

For the wavelength of 300 nm used, the constant is equal to 0.2874.

The contents were measured at 0, 5, 10, 15, 20, and 30 min.

The emulsions were tested in triplicate, and an ANOVA test was performed.

### 4.8. RNA Isolation and Transcriptomics

Since we reported and proved the occurrence of MAAs in our stressed cultures with UV–Vis spectroscopy and HPLC, the analysis continues with the isolation of the total mRNAs of each culture, their sequencing, and comparison via bioinformatics.

For the isolation of the total *J. luteoviridis* RNA of each control and treated culture, 300 mL of culture in the steady growth phase with an OD at 600 nm equal to 3 was centrifuged at 4000 rpm for 4 min so as to harvest the wet and fresh microalgal biomass. These biomasses were kept briefly in −20 °C so as to be pestled to powder using liquid nitrogen. Cells were disrupted for 15 min with the addition of glass beads in a homogenizer (Disruptor Genie by Scientific Industries, Bohemia, New York, NY, USA) with lysis buffer. The RNA isolation was performed using the Spectrum Plant total RNA kit by Sigma Aldrich. RNA quality and quantity were evaluated via agarose gel electrophoresis 1% *w*/*v*.

RNA sequencing was conducted using Illumina platforms based on the mechanism of SBS (sequencing by synthesis). Messenger RNA was purified from total RNA using poly-T oligo-attached magnetic beads. After fragmentation, the first strand cDNA was synthesized using random hexamer primers, followed by the second strand cDNA synthesis using either dUTP for a directional library or dTTP for a non-directional library. The library was checked with Qubit and real-time PCR for quantification and a bioanalyzer for size distribution detection. Data Quality Control was performed for raw data (raw reads) in fastq format. In this step, clean data (clean reads) were obtained by removing reads containing adapters, reads containing ploy-N, and low-quality reads from raw data. At the same time, Q20, Q30, and GC content were calculated. All downstream analyses were based on clean data of high quality. The reference genome for *Chlorella vairiabilis* and gene model annotation files were downloaded from the genome website (ncbi_refseq_chlorella_variabilis_v_1_0_gcf_000147415_1) directly for mapping. The index of the reference genome was built using Hisat2 v2.0.5, and paired-end clean reads were aligned to the reference 3 genome using Hisat2 v2.0.5. 2.3 Novel transcript prediction. The mapped reads for each sample were assembled using StringTie (v1.3.3b) in a reference-based approach. StringTie uses a novel network flow algorithm as well as an optional de novo assembly step to assemble and quantify full-length transcripts representing multiple splice variants for each gene locus.

As for the quantification of gene expression level, FeatureCounts v1.5.0-p3 was used to count the read numbers mapped to each gene. Then, FPKM (Fragments Per Kilobase of transcript sequence per million base pairs) of each gene was calculated based on the length of the gene and read count mapped to this gene. Differential expression analysis of two conditions/groups (two biological replicates per condition) was performed using the DESeq2R package (1.20.0). The resulting *p*-values were adjusted using Benjamini and Hochberg’s approach for controlling the false discovery rate. Genes with an adjusted *p*-value ≤ 0.05 found by DESeq2 were assigned as differentially expressed. Gene Ontology (GO) enrichment analysis of differentially expressed genes was implemented using the cluster Profiler R package, which corrects for gene length bias. GO terms with corrected *p* values less than 0.05 were considered significantly enriched by differentially expressed genes. Data were stored in NCBI SRA Bioproject PRJNA1345915 under a one-year ban. Based on EggNOG-mapper annotations, we further extracted transcripts annotated with shikimate pathway enzymes (aroA–E, aroK/L, ARO1/AROM), SAM-dependent O-methyltransferases (EC 2.1.1.*), and other aromatic amino acid metabolism genes, then examined their differential expression across contrasts.

## 5. Conclusions

Some types of algae can develop mechanisms to protect themselves from ultraviolet radiation, although it is generally known that plants can be negatively affected by ultraviolet radiation. One of these is the production of secondary metabolites absorbing UV, called MAAs, which can help them capture and retain photons without leaving their cellular machinery exposed. The availability of various products from microalgae is considered a promising prospect for the pharmaceutical, chemical, and nutraceutical industries. As cell factories, the efficiency of the algae’s extraction and purification methods determines their final utilization. Application of abiotic stressors—salinity, heat, and UV—can enhance the efficiency of MAA production in microalgae by activating stress-responsive biosynthetic pathways. Consistent with our results, using these stimuli to induce MAA accumulation suggests that the underlying genes are differentially expressed in a condition-specific manner. Optimization of culture parameters (stress intensity and duration, temperature, and light regime) together with stress selection led to a significant increase in MAA yield. The sustainable approach adopted in this study is noteworthy, as the application of mild heat stress did not result in the severe biomass loss observed under salinity or UV stress conditions. This study also revealed that extracts from *J. luteoviridis* can provide promising cosmeceutical applications in the new era of green and safe bioactive ingredients. These extracts, specifically the ones resulting from heat stress, are rich in MAAs and exhibit anti-aging, anti-inflammatory, and antioxidant properties. Furthermore, they showed enhanced ability to absorb UV and an increased SPF index when added to relative creams. The transcriptome of *J. luteoviridis* under four different growth conditions was sequenced using Illumina sequencing and analyzed in this study. Using a reference genome as a first approach, differential gene expression analysis was conducted to compare the different growth conditions. Our findings will facilitate investigations into *J. luteoviridis* and other Chorophyta based on the transcriptome to set the molecular basis of these secondary metabolites’ biosynthesis in various abiotic stresses. All stress treatments (UV, salinity, and heat) produced a substantial increase in peak intensity at 323–350 nm, whereas the control samples showed significantly lower absorption in this region. We also optimized an MAA extraction protocol suitable for “green” downstream applications in the pharmaceutical, nutraceutical, and cosmeceutical sectors and formulated an emulsion that showed preliminary positive results and exhibited an increased SPF index from 3.60 (control) to 3.78 when 0.2% MAA extract was added. Furthermore, we identified gene expression and overexpression of specific genes in the MAA pathway, like ArioC, AroM/Aro1 SAM methyltransferase, which could be targets for engineering enhanced MAA production.

## Figures and Tables

**Table 1:** SPF factor according to Mansur equation for the emulsion supplemented with MAAs.

Samples	Mansur Equation	SPF
Sunscreen cream SPF 20 BASE	3.61	3.60 ± 0.01
Sunscreen cream SPF 20 BASE (MAA 0.1%)	3.65	3.65 ± 0.03
Sunscreen cream SPF 20 BASE (MAA 0.2%)	3.78	3.78 ± 0.19

**Table 2:** Results of GO enrichment analysis of differential genes.

GOID	Description	*p*_value_	*p*_adj_	Gene ID	Gene Name	Up	Up_Gene_id	Down	Down_Gene_id
GO:0006412	translation	0.44	0.64	17357114/17356507/17355620	CHLNCDRAFT_59621/CHLNCDRAFT_34947/CHLNCDRAFT_59661	1	17355620	2	17357114/17356507
GO:0006518	peptide metabolic process	0.44	0.64	17357114/17356507/17355620	CHLNCDRAFT_59621/CHLNCDRAFT_34947/CHLNCDRAFT_59661	1	17355620	2	17357114/17356507
GO:0006807	nitrogen compound metabolic process	0.44	0.64	17357114/17356507/17355620	CHLNCDRAFT_59621/CHLNCDRAFT_34947/CHLNCDRAFT_59661	1	17355620	2	17357114/17356507
GO:0009059	macromolecule biosynthetic process	0.44	0.64	17357114/17356507/17355620	CHLNCDRAFT_59621/CHLNCDRAFT_34947/CHLNCDRAFT_59661	1	17355620	2	17357114/17356507
GO:0010467	gene expression	0.44	0.64	17357114/17356507/17355620	CHLNCDRAFT_59621/CHLNCDRAFT_34947/CHLNCDRAFT_59661	1	17355620	2	17357114/17356507

## Data Availability

The original data presented in the study are openly available in the NCBI sequence read archive (SRA) database under PRJNA1345915, and they are under review with a one-year embargo.

## Supplementary Materials

The following supporting information can be downloaded at: https://www.mdpi.com/article/10.3390/ijms27010537/s1.

## Abbreviations

The following abbreviation is used in this manuscript:

HPLC	High-Presure Liquid Chromatography.
