# Supplementary material for: Biosynthesis of UV-Absorbing Mycosporine-like Amino Acids and Transcriptomic Profiling of Differential Gene Expression in Green Microalga Under Abiotic Stresses
Source: Int J Mol Sci. 2026 Jan 5;27(1):537. doi: 10.3390/ijms27010537 (PMC12786583; doi:10.3390/ijms27010537)
Supplement: Supplementary file 1 [file ijms-27-00537-s001.zip › ijms-3983633-supplementary.pdf]

Supplementary Information:

Biosynthesis of UV-Absorbing Mycosporine-Like Amino Acids and Transcriptomic Profiling of Differential Gene Expression in Green Microalga Under Abiotic Stresses

Georgia Tsintzou [1], Evmorfia Bataka [2], Georgia Tagkalaki [3], Sofoklis Keisaris [4], Nikolaos Tsiropoulos [5], Nikolaos Labrou [6] and Panagiotis Madesis [1,4,*]

1 Laboratory of Molecular Biology of Plants, Department of Agriculture Crop Production and Rural Environment, School of Agricultural Sciences, University of Thessaly,38446 Volos, Greece
2 Laboratory of Biometry. Department of Agriculture Crop Production and Rural Environment, School of Agricultural Sciences, University of Thessaly,38446 Volos, Greece
3 Fresh Formula Private Limited Cosmetics Manufacturing Company, 1st km Lavriou Ave Koropiou—Markopoulou, 19400 Koropi, Greece
4 Institute of Applied Biosciences, Centre for Research and Technology, 57001 Thessaloniki, Greece
5 Laboratory of Analytical Chemistry and Agricultural Pharmacology, Department of Agriculture Crop Production and Rural Environment, School of Agricultural Sciences, University of Thessaly,38446 Volos, Greece
6 Laboratory of Enzyme Technology, Department of Biotechnology, School of Food, Biotechnology and Development, Agricultural University of Athens, 11855, Athens, Greece

*Correspondence author: Madesis P., Orcid, 0000-0003-1447-3514, e-mail: pmadesis@uth.gr,

....

Supplemental Table S1: Growth results based on Optical Density measurements at 600nm (OD600) for both controls and treated cultures in biological triplicates.

Treatment	OD600	Day		Treatment	OD600	Day
Control	2.56	0		NaCl Stress	2.57	0
Control	2.59	0		NaCl Stress	2.5	0
Control	2.61	0		NaCl Stress	2.55	0
Control	2.63	3		NaCl Stress	1.85	3
Control	2.69	3		NaCl Stress	1.9	3
Control	2.88	3		NaCl Stress	1.88	3
UV radiation	2.19	0		Heat Stress	2.41	0
UV radiation	2.24	0		Heat Stress	2.56	0
UV radiation	2.13	0		Heat Stress	2.54	0
UV radiation	1.64	3		Heat Stress	3.01	3
UV radiation	1.88	3		Heat Stress	3	3
UV radiation	1.15	3		Heat Stress	2.98	3

Supplemental Table S2: ANOVA table.

	Sum of Squares	Df	Mean Square	F	P-Value
Treatment	2.5153	3	0.8384	96.65	<0.001
Residuals	0.0694	8	0.0087		

Supplemental Table S3: ANOVA table

	term estimate std.error t-value p.value				
1	(Intercept)	0.147	0.054	2.727	0.026
2	treatmentHeat Stress	0.347	0.076	4.559	0.002
3	treatmentNaCl Stress	-0.810	0.076	-10.651	0.000
4	treatmentUV radiation	-0.577	0.076	-7.583	0.000

Supplemental Table S4: Post-Hoc LSD

	meas	groups
Heat Stress	0.493	a
Control	0.147	b
UV radiation	-0.430	c
NaCl Stress	-0.663	d

Supplemental Table S5: Post-Hoc LSD. Difference

	Difference (95% CI)	Adjusted p-value
Heat Stress-Control	0.347 (0.103,0.590)	0.008
NaCl Stress-Control	-0.810 (-1.054,-0.566)	0.000
UV radiation-Control	-0.577 (-0.820, -0.333)	0.000
NaCl Stress-Heat Stress	-1.157 (-1.400, -0.913)	0.000
UV radiation-Heat Stress	-0.923 (-1.167, -0.680)	0.000
UV radiation-NaCl Stress	0.233 (-0.010, 0.477)	0.060

....

Supplementary Table S6. Spectral index values [ABS(λ_{max})/ABS_{450}] calculated for cultures of *Jaagichlorella luteoviridis* under control and stress conditions. The ratio highlights the relative prominence of the MAA-like absorption band compared to background absorbance at 450 nm. Increased index values observed under UV, salinity, and heat stress indicate enhanced expression of MAA-type UV-absorbing compounds relative to the control.

Condition/λ_{max}	$ABS_{\lambda max}$/ABS_{450}
Control (312 nm)	2.00
UV-stressed (318 nm)	2.33
NaCl (salt-stressed) (334 nm)	2.33
Heat-stressed (339 nm)	5.33

Supplemental Table S7. Stability Test for 0,1% (w/w) MAA emulsion.	SUNSCREEN CREAM SPF20 MAA (0.1%)				
**3 MONTHS STABILITY TEST**					
**PARAMETER**	**3 Months**	**3 Months**	**3 Months**	**3 Months**	**3 Months**
**TEMPERATURE**	RT	5°C	40°C	45°C	50°C
**DATE**	4/3/2025	4/3/2025	4/3/2025	4/3/2025	4/3/2025
**Appearance**	OK	OK	OK	OK	Some oil on the surface
**Colour**	OK	OK	OK	OK	darker
**Odour**	OK	OK	OK	OK	OK
**pH (RT)**	6.01	6.02	5.95	5.90	5.86
**Viscosity (RT) ***	123.300cP 26.3%	129.800cP 27.7%	122.800cP 26.2%	120.500CP 25.7%	110.200CP 23.5%
**Centrifuge ****	1h: OK 2h:OK	1h: OK 2h:OK	1h: OK 2h:OK	1h: Ελάχιστο λάδι 2h:Ελάχιστο λάδι	1h: minor oil 2h:minor oil
**Microscope surface**	OK	OK	OK	OK	Detaches

Supplemental Table S8. Stability Test for 0.2% (w/w) MAA emulsion

	SUNSCREEN CREAM SPF20 MAA (0.2%)				
**3 MONTHS STABILITY TEST**					
**PARAMETER**	**3 Months**	**3 Months**	**3 Months**	**3 Months**	**3 Months**
**TEMPERATURE**	RT	5°C	40°C	45°C	50°C
**Appearance**	OK	OK	OK	OK	Some oil on the surface
**Colour**	OK	OK	OK	OK	darker
**Odour**	OK	OK	OK	OK	OK
**pH (RT)**	6.09	6.10	6.04	5.92	5.88
**Viscosity (RT)***	109.200cP 23.3%	112.000cP 23.9%	110.200cP 23.5%	107.30cP 22.98%	100.800cP 21.5%
**Centrifuge****	1h: OK 2h:OK	1h: OK 2h: OK	1h: OK 2h:OK	1h: oil release was negligible 2h: oil release was negligible	1h: oil release was negligible 2h: oil release was negligible
**Microscope surface**	OK	OK	OK	Minor phase separation	Minor phase separation

**REMARKS**	* (cP) Brookfield RVT. Spindle. Speed:	S95/1rpm
	* 20°C for Shampoo and Wash	
	** 1h 3500 rpm and afterwards 1h 3500 rpm	

Supplementary figure S1

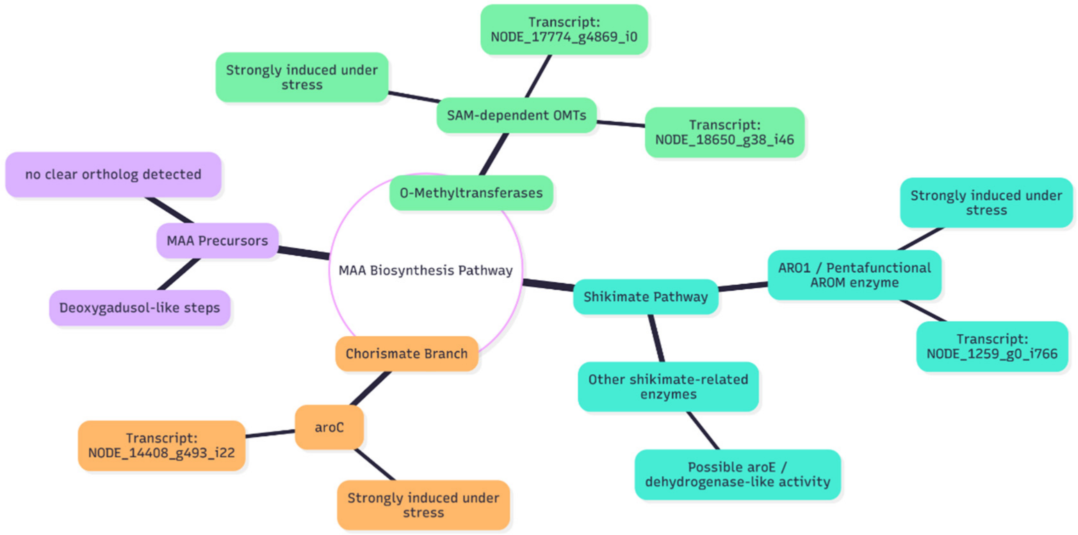

Supplementary Figure 1 Concept map summarizing candidate genes and metabolic bran Jaagichlorella. The diagram highlights three major modules—precursor formation, the shikimate/chorismate pathway, and O-methyltransferases (OMTs)—including putative transcripts and notes on expression patterns under stress. Unknown steps and missing orthologs are indicated where biosynthetic links remain unresolved .
